# Gastric-type adenocarcinoma of the uterine cervix: clinical features and future directions

**DOI:** 10.1093/jjco/hyae019

**Published:** 2024-02-15

**Authors:** Hiroshi Nishio, Risa Matsuda, Takashi Iwata, Wataru Yamagami

**Affiliations:** Department of Obstetrics and Gynecology, Keio University School of Medicine, Tokyo, Japan; Department of Obstetrics and Gynecology, Keio University School of Medicine, Tokyo, Japan; Department of Obstetrics and Gynecology, Keio University School of Medicine, Tokyo, Japan; Department of Obstetrics and Gynecology, Keio University School of Medicine, Tokyo, Japan

**Keywords:** gastric-type adenocarcinoma (GAS), HPV-independent, gene mutation

## Abstract

The concept of gastric-type mucinous carcinoma of the uterine cervix (GAS) has been accepted worldwide because of its aggressive clinical behaviour and the absence of high-risk human papilloma virus infection. The World Health Organization (WHO) 2020 classification divides cervical tumours into two categories: human papilloma virus-associated and human papilloma virus-independent. Hence, GAS is now classified as an human papilloma virus-independent gastric type. Because clinical studies have reported that GAS is refractory to conventional treatments such as chemotherapy and radiotherapy, especially at an advanced stage, and has aggressive features with widespread dissemination to unusual sites, such as the omentum, peritoneum and distant organs, it is urgent to establish new treatment strategies by comparing the molecular profiles of human papilloma virus-associated adenocarcinomas. A series of genetic mutations characteristic to GAS encourage the development of future treatment strategies such as targeted therapy and immunotherapy.

## Teaser

The gastric-type mucinous carcinoma of the uterine cervix (GAS) is clinically poor histological type, which has a series of specific genetic mutations. Future studies will develop effective treatments based on patients’ genetic mutational backgrounds.

## Introduction

Cervical cancer is the fourth most common cancer in women, accounting for 570 000 new cancer cases worldwide and 311 000 deaths annually (1). In recent decades, non-HPV-associated cervical adenocarcinomas have represented a growing proportion of new diagnoses (2), likely due in part to improved screening programmes as well as increased uptake of human papillomavirus (HPV) vaccination (3). Histological-type adenocarcinomas now account for 25% of newly diagnosed cervical cancer cases (4,5), with gastric-type adenocarcinoma (GAS) comprising 10% of all newly diagnosed cervical adenocarcinomas (6).

GAS is a rare histological subtype of cervical adenocarcinoma that is not secondary to HPV infection (5). GAS is morphologically distinguished by tumour cells with distinct cell borders and voluminous pale eosinophilic cytoplasm, which was initially described by Kojima et al., accounting for nearly 20% of all cervical adenocarcinoma cases in Japan (7,8). GAS represents a spectrum of histological findings, from the bland appearance of minimal deviation adenocarcinoma (MDA) to more dysplastic changes. MDA, previously described as ‘adenoma malignum’, is a deceptively well-differentiated form of GAS that is nearly indistinguishable from normal glands, making diagnosis on cytology and biopsy difficult (5,9,10). In addition to its low detection rate by screening, GAS frequently locates within a high lesion in the endocervical canal, with a bulky, infiltrative mass (11) usually detected by multimodal imaging studies. GAS classically arises in middle-aged women with symptoms of watery vaginal discharge and abnormal uterine bleeding. Because of its difficulty of diagnosis, it is not surprising that more than 50% of patients have stage II or greater disease at presentation (7). When matched for stage with patients with usual-type endocervical adenocarcinoma (UEA), patients with GAS have worse overall survival (OS) and disease-specific survival according to the three different retrospective analyses (5). Eighty percent of GAS cases are well differentiated, whereas ~60% of UEA cases are well differentiated (12). Precursor lesions are considered to include atypical lobular endocervical glandular hyperplasia (LEGH) and GAS *in situ* (13). Moreover, the association with Peutz–Jeghers syndrome comprising *STK11* variant is well known, as well as the association with bilateral ovarian mucinous neoplasms or sex cord tumours.

GAS has recently been identified as a distinct entity with WHO classification, but how it should be clinically managed is still unclear because of its rarity. Treatment paradigms for cervical cancer have primarily been driven by data from HPV-associated diseases, and it is unknown whether patients with GAS derive the same clinical benefit from these approaches. Although uncommon, GAS is considered to increase in relative prevalence with the introduction of HPV vaccination and precursor lesions will not be detected by primary HPV-based screening. In this review, we discuss the clinical findings of GAS and outline the molecular biological features identified in recent studies. We further assess future therapeutic strategies.

## Clinical pathological features

### WHO classification and pathological overview

Until the late 2000s, gynaecological pathologists and oncologists considered that cervical adenocarcinoma was an HPV-derived single disease in terms of its aetiology, pathogenesis and biology, which were mucinous, endometrioid and serous carcinoma. However, this classification has been changed because endocervical adenocarcinoma is now considered to comprise a heterogenous neoplasm and is divided by HPV association status (14). In the WHO 2020 classification, new categories of HPV-associated and HPV-independent cervical adenocarcinomas were defined. GAS is classified as an HPV-independent gastric type, whereas clear cell carcinoma, mesonephric carcinoma and endometrioid carcinoma are classified as other adenocarcinomas (ADCs) of the uterine cervix (15). Minimal deviation adenocarcinoma (MDA) is a rare subtype of mucinous ADC, accounting for only 1–3% of all cervical ADCs; however, this particular type of ADC came to prominence in 1998 when it was reported to have gastro-pyloric gland traits (10). Subsequently, it became clear that some tumours conventionally diagnosed as endocervical-type mucinous ADC also have gastric-type traits (16). In contrast to histology of UEA, which contains elongated and pseudostratified hyperchromatic nuclei, GAS is defined as tumour-containing cells that show gastric differentiation with abundant clear or pale eosinophilic cytoplasm, distinct cytoplasmic borders, a low nuclear-to-cytoplasmic ratio and basally located nuclei (17). MDA, which is included in the spectrum of GAS, is characterized by a low-grade morphology, with well-differentiated glands that sometimes show a ‘claw-like’ proliferation pattern, lined by cells with abundant intracytoplasmic mucin and minimally atypical nuclei (14). The glands are randomly distributed within the stroma, sometimes with minimal or no desmoplasia (18). GAS can be diagnosed with high reproducibility but sometimes requires analysis of diagnostic markers by immunohistochemistry (7,19). GAS has a wide range of atypia, from minimal to marked. The tumour cells form glands and solid areas, but papillary, trabecular or single-cell patterns can be present (20). The glands deeply infiltrate stroma, including a desmoplastic response, and lymphovascular invasion is also common.

### Diagnostic markers

GAS produces pyloric-type mucin that stains pink or red on Alcian blue histochemical staining, whereas normal endocervical mucins are acidic and stain dark blue. We need to be aware that the criteria of a combination of multiple cysts on imaging and gastric immunophenotype shown by HK1083 (M-GGMC-1) IHC and/or Periodic Acid-Schiff (PAS) reaction do not necessarily support a diagnosis of GAS. GAS morphologically resembles ADCs of the pancreas to the biliary tract, with a similar immunohistochemical profile; one difference is that most GAS cases express PAX8 with diffuse or focal positivity (21). PAX8 expression is positive in 68–80% of GAS, which is useful in distinguishing these tumours from ADCs of gastrointestinal or pancreas to biliary origin. However, PAX2 expression is typically negative. Gastric-type mucin markers, such as MUC6 or HIK1083 (10), are present in 60–80% of these carcinomas, and MUC6 can be expressed in other ADC tumour types (7,21–23). GAS is usually negative or shows patchy, non-block-type immunoreactivity with p16, which is a well-known marker for HPV infection; however, occasional examples exhibit block-type immunoreactivity with p16 (17,12,21). These carcinomas show aberrant (mutation-type) expression of p53 in up to 50% of cases (22). Estrogen receptor (ER), progesterone receptor (PR), vimentin, p63, p40 and androgen receptor (AR) expression is usually negative (21,22). GAS is positive for trefoil factor 2 (TFF2) (24), CK7, CEA and CA-IX, and up to 50% of cases can be positive for CK20 and CDX2 (21,22). Interestingly, HNF1β, which is a marker for clear cell histology, can be positive in up to 90% of cases, whereas Napsin A may be positive in a much smaller percentage of cases, which can be valuable diagnostic marker for distinguishing GAS from clear cell carcinoma (22,25).

When GAS is difficult to distinguish from UEA, the presence of prominent mitotic and apoptotic activities favour a UEA diagnosis. As we mentioned, testing for MUC6, HIK1083, p16 and HPV status may be valuable. High-grade squamous intraepithelial lesions and HPV-associated ADC *in situ* favour a usual-type diagnosis, although small numbers of GAS cases may contain such lesions coincidentally.

The precursor lesions of GAS are atypical LEGH or gastric-type adenocarcinoma *in situ*, which favour a gastric-type diagnosis. Immunohistochemistry (IHC) is of limited value in distinguishing between LEGH and well-differentiated variants of GAS, emphasizing the value of haematoxylin and eosin staining to separate these entities. Interestingly, carcinomas exhibiting an overlapping morphology with areas resembling both UEA and GAS have been described. In these difficult cases, HPV and p16 testing can usually identify whether it is an HPV-associated mucinous carcinoma or GAS with morphological similarity, rather than a true mixed carcinoma, which likely does not occur (26). As mentioned above, it may be particularly difficult to differentiate GAS from clear cell carcinoma, especially with biopsy materials, because HNF1β and Napsin A can be positive in both tumours, although Napsin A has usually lower positivity (25). A combination of HIK1083 and TFF2 testing can be useful because TFF2 is expressed in 80% of GAS cases compared with 12% of non-GAS cases, with no clear cell carcinomas showing positive results. Dual positivity for TFF2 and HIK1083 is highly specific for GAS (27). When distinguishing between MDA and various benign cervical glandular lesions such as Nabothian cysts or endocervical glandular hyperplasia, ER/PR staining can be useful because GAS cases are usually negative and mostly have benign glandular lesions. The claw-like shape and deep placement of glands, along with mild nuclear atypia and the presence of focal stromal desmoplasia, are also more typical of GAS than various benign cervical glandular lesions. These features are helpful in distinguishing GAS from LEGH, in which a preserved lobular architecture with minimal cytologic atypia is present. Some authors have suggested that smooth muscle actin (SMA) staining may be helpful because this marker is positive in cervical stromal cells adjacent to the invasive glands of GAS and negative in stromal cells surrounding LEGH (28). GAS, including MDA, exhibits aberrant/mutation p53 staining in up to 50% of cases, whereas LEGH exhibits wild-type immunoreactivity. PAX2 expression is negative in GAS and positive in LEGH (21,29).

Somatic copy number gain of *APOBEC3B* is common in GAS but rare in UEA. According to the Chinese study done by Liao et al., enrichment of the APOBEC signature was attributed to the frequent gain of somatic copy number alteration of *APOBEC3B* (20%), which perfectly matched the nuclear-positive staining of APOBEC3B through IHC. In contrast, *APOBEC3B* alterations were absent in patients with UEA. Notably, positive APOBEC3B was consistently enriched in patients with a favourable prognosis in both the discovery cohort and additional patients with GAS (30). Thus, positive IHC staining for APOBEC3B can serve as a potential biomarker for the favourable prognosis of GAS (30).

Another cell surface marker, Claudin-18, was expressed significantly more frequently in GAS than in non-GAS (Müllerian or intestinal immunophenotypes) (31). In other types of ADCs with tissue microarrays, Claudin-18 expression was significantly higher in GAS (65%) than in UEA (2%) (*P* < 0.01) with tissue microarrays (32). All claudin-18-positive GAS cases showed intense staining, in 21 of 22 cases. Claudin-18 shared the same degree of sensitivity and specificity as HIK1083 and TFF2. Claudin-18 with intense staining can be a promising surrogate marker for distinguishing GAS from other types of ADCs, including clear cell carcinoma (32).

### Treatment outcome of GAS

GAS is more common in patients in their 40s and is not reported to differ significantly from UEA in terms of age at onset, with variation among reports (31,33). For the last several decades, all ADCs were treated in a similar fashion, and histopathological diagnosis did not have any impact on patient management. The involvement of high-risk HPV is well known in most cases of UEAs but is not necessarily universal. In previous studies, the rate of detection of high-risk HPV among adenocarcinomas varies significantly, from 60% to almost 100%, presumably reflecting differences in detection assays, types of specimens, population and geography. Some studies have reported that adenocarcinomas in the same cohorts are less frequently positive for high-risk HPV than squamous cell carcinoma (SCC) (34,35). However, mutant-pattern p53 immunoreactivity has been reported to be an adverse prognostic factor in adenocarcinoma, and such tumours are commonly negative for high-risk HPV (36,37). Differences in patient outcome between SCC and ADCs are also a controversial issue (38). Although National Comprehensive Cancer Network (NCCN) guidelines do not distinguish between SCC and ADCs, others have suggested an unfavourable prognosis among patients with ADCs, with a survival rate 10–20% lower than that for cases with SCC (39). Lymph node metastasis, ovarian metastasis, peritoneal spread and resistance to radiotherapy are more common in cases of ADCs (40,41), which are specific features of GAS. It is conceivable that these differences are due to the existence of HPV-independent adenocarcinoma, which shows *p53* variants and may have an impact on the prognosis of patients with ADCs as a whole. This hypothesis is strengthened by the concept of GAS.

Previous studies have shown that GAS cases were refractory to conventional treatments. Among GAS patients who develop recurrent diseases, 40–60% of patients have pelvic locoregional recurrences, the majority of which occur in the first year after completion of their first treatment (33,42). According to the previous study reported by Nishio et al. (42), patients with GAS demonstrated an inferior response rate to the radiation therapy compared with patients with UEA (50% vs 82%, respectively, *P* < 0.0001). GAS could demonstrate less sensitivity to chemotherapy compared with HPV-associated carcinomas. Kojima et al. (43) reported that the pathologic response rate of GAS cases to neoadjuvant chemotherapy with docetaxel and carboplatin was significantly lower than that of patients with UEA.

### Genomic analysis of GAS

The Cancer Genome Atlas database showed that in cervical cancer, HPV-independent cases had higher epithelial–mesenchymal transition (EMT) messenger RNA scores and significantly lower promoter methylation levels compared with HPV-associated patients (44). The genomic profiles of GAS identified previously were basically consistent across the studies, including alterations in cell cycle genes such as *TP53* and *CDKN2A*, as well as *KRAS* hotspot variants, which were the most common (45,46). Garg et al. performed a genetic analysis to assess 161 unique cancer-driver genes for GAS and found 92 variants from 14 samples. *TP53* was the most recurrently observed variant, followed by *MSH6*, *CDKN2A/B*, *POLE*, *SLX4*, *ARID1A*, *STK11*, *BRCA2* and *MSH2.* These genetic variants were related to DNA damage repair, cell cycle, the Fanconi anaemia pathway and the PIK3CA–AKT pathway. Gene variants found in known endometrial cancers, such as *POLE*, *ARID1A*, *KRAS* and *FBXW7*, were also found in GAS (47)*.* Another study was undertaken by Park et al. in 21 GAS cases with a customized panel containing 94 cancer-related genes. A total of 54 somatic variants were detected, and the most frequently mutated gene was *TP53* (52%), followed by *STK11*, *HLA-B*, *PTPRS* (all 19.9%), *FGFR4* (14.3%), and *GNAS*, *BRCA2*, *ELF3*, *ERBB3*, *KMT2D* and *SLX4* (9.5%). These variants are related to DNA damage repair and EMT. They concluded that EMT pathways may be involved in the aggressive nature of GAS chemoresistance (45). Up to 10% of GAS cases are associated with Peutz–Jeghers syndrome, which is caused by a germline mutation in the *STK11/LKB1* gene located on chromosome 19p13.3 (48). In non-small cell lung cancer, *STK11/LKB1* mutation is associated with a shorter OS compared with wild-type (49). *STK11/LKB1* mutations are considered to be associated with PD-1 inhibitor resistance in *KRAS*-mutant lung adenocarcinoma (50).

Alterations were seen in *ERBB2* and *PIK3CA* that may be potentially targetable in this rare disease (46). Approximately 5–15% of GAS cases are *ERBB2* amplified, as defined by dual colour *in situ* hybridization and immunohistochemistry (51,52). Ehmann et al. reported *ERBB2*-altered GAS cases were treated with the anti-HER2 antibody trastuzumab with excellent clinical outcomes, consistent with the experience in other HER2-positive cancers such as endometrial, breast and gastric cancer (33,52,53). DESTINY-PanTumor02 evaluated the efficacy of trastuzumab deruxtecan in patients with various cancer types who had failed standard treatment. A total of 120 patients with gynaecologic malignancies were included, in which objective response rate (ORR) among patients with cervical cancer was 50.0%. The ORR was particularly high in the HER2+ and HER3+ groups, suggesting that this treatment can be an option for HER2-positive GAS cases (54). This may offer a new treatment paradigm for this small subset of patients with GAS and should be explored further in prospective studies including antibody–drug conjugates as in non-small cell lung cancer or breast cancer. KRAS may be targeted using sotorasib and other KRAS p.G12C inhibitors (55); this may offer another therapeutic opportunity.

As mentioned above, GAS resembles pancreato-biliary tumours in both histology and genomic drivers (6) because it is enriched with alterations in *KRAS*, *TP53*, *SMAD4* and *CDKN2A* (4,56). GAS is also similar to intestinal-type gastric adenocarcinoma, sharing gene alterations in *KMT2D*, *ERBB3* and *RNF43*, which have been associated with poor prognosis (45). Given these similarities, consideration should be given to prospectively evaluating gastrointestinal cancer regimens such as those incorporating 5-fluorouracil for the treatment of GAS, either upfront or in the recurrent setting. In addition, because the genomic alterations observed in GAS such as *STK11/LKB1* have been well annotated in other solid tumour types, they may represent prognostic or predictive biomarkers. Larger prospective studies are warranted to assess whether the presence of specific genetic alterations is associated with outcome in GAS.

## Conclusion

As reviewed in this article, GAS has been confirmed to be poor, even in cases of early-stage cancer and following surgical resection. Because of its clinical features, GAS is usually diagnosed at a late stage and has generally poor outcomes compared with UEA at the same stage.

Recent studies have elucidated several molecular and biological features of GAS. Next Generation Sequencing (NGS) analysis revealed that *TP53* mutations were the most common, accounting for nearly half of all cases, followed by *KRAS*, *ARID1A*, *PTEN*, *CDKN2A* and *STK11* variants. All of these have been reported in gastrointestinal and pancreatic cancers. The similarity of the genetic backgrounds of these mutations in gastrointestinal, pancreatic, biliary tract and endometrial cancer may help in the development of therapeutic strategies. Moreover, *ERBB2* amplification has been a remarkable potential target gene for molecular-targeted drugs and has been found among several groups of patients with GAS. Future studies will develop effective treatments for GAS based on patients’ genetic mutational backgrounds.
